# Employment trajectories and biological aging: Racial and ethnic disparities in work instability and epigenetic age acceleration

**DOI:** 10.1016/j.ssmph.2026.101973

**Published:** 2026-09-23

**Authors:** Wen-Hua Lai, Ting-Syuan Lin, Tsai-Chin Cho, Helen C.S. Meier, Farah Ammous, Amanda N. Leggett

**Affiliations:** a Irsay Institute, Indiana University, Bloomington, USA; b Department of Sociology, University of Illinois Urbana-Champaign, USA; c Department of Epidemiology, University of Michigan School of Public Health, USA; d Center for Social Epidemiology and Population Health, University of Michigan School of Public Health, USA; e Survey Research Center, Institute for Social Research, University of Michigan, USA; f School of Kinesiology, University of Michigan, USA; g Institute of Gerontology, Wayne State University, USA; h Department of Psychology, Wayne State University, USA

**Keywords:** Employment trajectories, Epigenetic age acceleration, Biological aging, Racial and ethnic disparities, Weathering hypothesis, Life course

## Abstract

Racial and ethnic inequalities in healthy aging may reflect the cumulative biological toll of structural disadvantage. Employment is one life-course pathway through which this disadvantage is reproduced, yet most research relies on point-in-time measures rather than long-term work histories. Guided by the weathering hypothesis, this study examined whether life-course employment trajectories were patterned by race and ethnicity, whether they were associated with epigenetic age acceleration (EAA), and whether racial and ethnic differences in EAA persisted within similar work histories. Using the 2016 Health and Retirement Study with the linked Life History Mail Survey and Venous Blood Study, we constructed employment histories from ages 18 to 50 and applied sequence and cluster analysis to identify four trajectories. Multinomial logistic regression showed that Black and Hispanic adults had more than twice the odds of “Late Workforce Entry” than White adults, with differences concentrated in delayed entry rather than mid-career disruption or non-employment. In survey-weighted OLS models across five epigenetic clocks (N = 1861), employment trajectory was largely unrelated to EAA and did not moderate racial and ethnic differences in it. Racial differences nonetheless persisted within trajectories: Black adults showed faster DunedinPACE and GrimAge acceleration than White adults, with DunedinPACE differences within stable full-time work and both differences within long-term non-employment, while differing in the opposite direction on the age-predictive Hannum clock. These findings partially align with the weathering hypothesis by showing that racial differences in biological aging persist even within similar employment trajectories. This pattern suggests that the biological consequences of employment may depend not only on long-term work patterns, but also on the unequal conditions and experiences embedded within them.

## Highlights

• Life-course employment trajectories differed by race and ethnicity.
• Black and Hispanic adults more often had late-entry, less stable work trajectories.
• Work histories were not consistently linked to epigenetic aging.
• Black–White aging gaps persisted in stable full-time and long-term non-employment paths.
• Findings suggest unequal conditions within jobs drive biological aging differences.

## 1 Introduction

Racial and ethnic inequalities in healthy aging remain among the most persistent features of population health in the United States. Black adults experience earlier onset of chronic disease, physiological decline, and higher morbidity and mortality than White adults of similar age, while Hispanic adults carry elevated burdens of diabetes and disability within a more complex mortality pattern that varies by nativity (Geronimus et al., 2006; Markides & Eschbach, 2005). The weathering hypothesis argues that these disparities reflect the cumulative physiological toll of chronic stress rooted in structural disadvantage, systemic racism, and socioeconomic adversity across the life course (Geronimus et al., 2006). Epigenetic clocks offer one way to assess this biological embedding by estimating whether biological aging has outpaced chronological age, capturing morbidity- and mortality-relevant biological wear (Crimmins, 2020; Levine et al., 2018; Lu et al., 2019).

Employment is a central channel through which social inequality is reproduced and translated into health disparities, including disparities in aging (Benach et al., 2014; Farina et al., 2026; Lipscomb et al., 2006). Across adulthood, work structures income, health insurance, retirement security, social status, daily routines, cognitive and social engagement, and exposure to psychosocial stress and occupational hazards (Benach et al., 2014; Eisenberg-Guyot et al., 2020; Lipscomb et al., 2006). Prior research links adverse employment conditions to morbidity, mortality, and accelerated epigenetic aging, but much of this evidence relies on employment status or job characteristics measured at a single point in time (Andrasfay, Kim, et al., 2023; Benach et al., 2014; Farina et al., 2026; Friedland & Price, 2003; Lipscomb et al., 2006; Virtanen et al., 2005). These approaches cannot capture how work and non-work experiences accumulate, sequence, and change across adulthood (Wahrendorf et al., 2019).

A life-course perspective helps address this limitation by treating employment as a process that unfolds across adulthood rather than as a single status or job characteristic. Socially patterned differences in employment and socioeconomic disadvantage may accumulate over decades, widening health gaps as cohorts age (Dannefer, 2003; Ferraro & Shippee, 2009; Raffington & Belsky, 2022). Evidence on epigenetic aging supports this cumulative logic. Specifically, biological aging appears more strongly patterned by early-life and accumulated disadvantage than by socioeconomic position measured at a single point in adulthood (Austin et al., 2018; McCrory et al., 2019). A trajectory-based approach instead treats employment as a life-course process, allowing long-term patterns of labor-force attachment, disruption, and non-employment to be examined in relation to later-life biological aging.

This trajectory-based approach is especially important for studying racial and ethnic disparities because employment histories are racially patterned and may carry unequal meanings across groups. In the U.S., Black and Hispanic workers are more likely than White workers to experience delayed workforce entry, repeated employment disruption, and long-term non-employment rather than stable full-time careers (Delaporte, 2025; McDonough et al., 2017; Wahrendorf et al., 2019). Yet racial inequality operates not only through sorting into different trajectories, but also through unequal conditions within similar trajectories. Even when workers follow comparable patterns of labor-force attachment, Black and Hispanic workers are more likely to occupy lower-wage, less secure, and more hazardous jobs, with fewer benefits, weaker protections, lower job control and greater exposure to discrimination (Doede, 2016; Eisenberg-Guyot et al., 2020; Lipscomb et al., 2006; Mintz & Krymkowski, 2010). The same trajectory need not represent the same exposure across racial and ethnic groups. It therefore remains unclear whether racial and ethnic differences in biological aging are associated with unequal sorting into life-course employment pathways, or whether they persist among adults with similar work histories.

Guided by a life-course framework and the weathering hypothesis, this study examines employment trajectories as socially patterned pathways through which racialized labor-market disadvantage may become biologically embedded. Using the Health and Retirement Study (HRS), we link sequence-based employment trajectories from ages 18 to 50 with five epigenetic clocks: Horvath, Hannum, PhenoAge, GrimAge, and DunedinPACE. We ask whether employment trajectories are racially and ethnically patterned (RQ1), whether they are associated with EAA (RQ2), whether they modify racial and ethnic differences in EAA (RQ3), and whether racial and ethnic differences in EAA persist within trajectories among adults with similar work histories (RQ4). Because epigenetic aging is measured cross-sectionally and the clocks capture different dimensions of aging, we treat these analyses as descriptive and exploratory rather than as tests of causal mechanisms.

## 2 Background

### 2.1 Weathering, racialized employment, and unequal exposure

The weathering hypothesis provides a framework for interpreting racial and ethnic differences in biological aging as the embodied consequence of accumulated racialized disadvantage (Forde et al., 2019; Geronimus et al., 2006). Weathering conceptualizes greater chronic exposure to economic insecurity, discrimination, residential and occupational hazards, and other forms of chronic stress as a process that accelerates normal aging among disadvantaged groups (Geronimus et al., 2006; Simons et al., 2021). Over time, these exposures may become biologically embedded through physiological wear across stress-related neuroendocrine, inflammatory, and immune systems (Doede, 2016; Geronimus et al., 2006; Lipscomb et al., 2006; McEwen & Seeman, 1999). A systematic review found evidence that weathering contributes to racial disparities across multiple aging-related outcomes and biological mechanisms, including hypertension, diabetes, stroke, functional limitations, longevity, self-rated health, allostatic load, telomere length, inflammation, and DNA methylation-based aging measures (Forde et al., 2019).

Applied to employment, weathering suggests that racialized labor-market conditions may contribute to biological aging by shaping both access to stable work and the exposures attached to similar work histories (Doede, 2016; Lipscomb et al., 2006). Racial/ethnic inequality may therefore operate not only through unequal sorting into employment trajectories (Eisenberg-Guyot et al., 2020), but also through unequal exposure within similar trajectories (Assari, 2025). Consistent with a diminished-returns perspective, higher-status work may confer weaker health benefits for Black than for White adults at similar levels of occupational standing (Assari, 2025), while job loss or precarious work may carry larger health penalties for Black workers (Burgard et al., 2009; Lipscomb et al., 2006). Thus, the same employment trajectory may not represent the same exposure, resources, or biological consequences across racial and ethnic groups. Whether racial and ethnic differences in epigenetic aging persist within similar employment trajectories remains unexamined.

### 2.2 Epigenetic aging as a marker of biological embedding

Epigenetic age, derived from DNA methylation patterns at specific CpG sites, estimates an individual's biological age (Liang et al., 2024; Raffington & Belsky, 2022; Simons et al., 2021). Epigenetic age acceleration (EAA) captures the gap between this estimate and chronological age, indicating whether the body has aged faster or slower than expected (Simons et al., 2021). Positive EAA has been associated with higher risk of frailty, cardiovascular disease, cognitive decline, and all-cause mortality, often before clinical disease becomes apparent (George et al., 2021; Robinson et al., 2020; Simons et al., 2021). Because DNA methylation is more responsive to chronic than acute exposures, EAA may reflect cumulative biological responses to chronic social and economic adversity (Crimmins, 2020; Schmitz et al., 2022; Simons et al., 2021; Willems et al., 2025). It therefore offers a useful marker for examining how racially patterned life-course disadvantage becomes biologically embedded, consistent with the weathering hypothesis (Crimmins, 2020; Simons et al., 2021; Willems et al., 2025).

Epigenetic clocks differ in what they are designed to capture (Liang et al., 2024; Raffington & Belsky, 2022; Simons et al., 2021). First-generation clocks, such as Horvath and Hannum, were developed primarily to predict chronological age and tend to be less strongly associated with morbidity and mortality (Crimmins, 2020; Raffington & Belsky, 2022; Schmitz et al., 2022; Simons et al., 2021; Willems et al., 2025), while second-generation clocks were designed to capture stress-relevant biological aging (Belsky et al., 2018; Lu et al., 2019; Simons et al., 2021). PhenoAge reflects physiological dysregulation and disease risk through clinical biomarkers, whereas GrimAge and DunedinPACE may more directly index the cumulative, long-term biological wear implied by weathering (Levine et al., 2018; Lu et al., 2019; Raffington & Belsky, 2022). Comparing multiple clocks therefore helps clarify whether racialized employment histories are associated broadly with epigenetic aging or more specifically with clocks tied to morbidity, mortality, and physiological decline.

### 2.3 Employment conditions, instability, and epigenetic aging

Evidence linking employment to epigenetic aging remains limited and has focused mainly on occupational type, work conditions, or employment status. Lower occupational prestige and employment in service, sales, clerical, manual, and unskilled occupations have been associated with faster epigenetic aging than professional and managerial work, particularly on GrimAge and DunedinPACE (Andrasfay, Crimmins, et al., 2023; Andrasfay, Kim, et al., 2023; Farina et al., 2026). Adverse work conditions, including low job control, night-shift work, and long working hours, have each been associated with roughly 1.4 to 2.1 years of accelerated aging (Andrasfay, Crimmins, et al., 2023). Evidence for employment instability is mixed: unemployment, underemployment, job insecurity, low wages, and night-shift work have been linked to acceleration across multiple clocks (Farina et al., 2023, 2026; Freni-sterrantino et al., 2022), whereas some studies find no association between unemployment duration and the Hannum or Horvath clocks (Hughes et al., 2018).

Two gaps remain. First, point-in-time employment measures leave the cumulative and sequenced nature of work histories underexamined (Wahrendorf et al., 2019). Second, although the health returns to similar occupations may differ by race (Assari, 2025), it remains unclear whether racial and ethnic differences in biological aging reflect sorting into different employment trajectories or unequal health returns to similar trajectories, that is, whether the same employment conditions confer weaker health benefits for racialized groups. Using nationally representative work-history data, this study assesses racial and ethnic differences in sequence-based employment trajectories across early and mid-adulthood, links these trajectories to later-life epigenetic aging across multiple clocks, and uses within-trajectory comparisons to examine whether racial and ethnic differences in biological aging persist among adults with similar employment trajectories.

## 3 Methods

### 3.1 Data & analytic sample

Data come from the Health and Retirement Study (HRS), a nationally representative longitudinal study of U.S. adults over age 50. The HRS oversamples Black and Hispanic respondents, supporting racial and ethnic comparisons. We linked the 2016 Core Survey, the 2015/2017 Life History Mail Survey (LHMS, n = 9453), and the Venous Blood Study (VBS) DNA methylation subsample (N = 4018). The DNA methylation sample was drawn from a non-random VBS subsample that, when weighted, represents the overall HRS sample; epigenetic age was measured in the 2016 VBS among respondents aged 55 and older. The LHMS collected retrospective education and employment histories, including job start and end dates and reasons for leaving up to ten prior jobs.

Among VBS respondents, 2334 had linked LHMS employment histories. We used two analytic samples. The sequence analysis sample included respondents with valid race/ethnicity classification, useable employment histories from ages 18 to 50, and valid HRS-VBS sampling weights. From the linked LHMS–VBS sample, we excluded respondents who identified as non-Hispanic Other race/ethnicity (n = 55), had incomplete employment sequences (n = 67), or lacked valid HRS-VBS sampling weights (n = 65), yielding a sequence sample of 2147 respondents. This sample was used to identify and describe employment trajectories. The regression sample further excluded respondents with missing covariates, including nativity (n = 286), yielding a final analytic sample of 1861 respondents. This sample was used for multinomial logistic models of trajectory membership and OLS models of epigenetic age acceleration. We used complete-case analysis because missingness was limited and concentrated in a small number of covariates. All analyses applied HRS-VBS subsample weights to account for the complex survey design. The sample derivation is shown in Supplementary Fig. S1.

### 3.2 Employment trajectories

The LHMS employment histories were converted into annual employment states from ages 18 to 50 using retrospective reports of full- and part-time employment, job duration, job transitions, and reasons for non-employment after completion of full-time education. This yielded a 33-year sequence of annual employment states for each respondent. Annual states were coded as follows: full-time employment, part-time employment, non-employment due to caregiving, non-employment due to unemployment, non-employment due to disability or medical leave, non-employment for other reasons, and years before the first job record. Because respondents varied in age at the 2016 assessment, extending trajectories beyond age 50 would give older respondents longer observed histories and more opportunities for employment transitions. We restricted the trajectory window to ages 18–50 to ensure comparable sequence length across respondents and to avoid later-life exits that may reflect retirement or health decline. Epigenetic age was measured after the trajectory window.

We used sequence analysis to identify long-term employment trajectories, treating each respondent's employment history as an ordered sequence of categorical states rather than as isolated employment statuses (Abbott & Tsay, 2000; Billari & Piccarreta, 2005). This approach captures the timing, duration, and ordering of employment and different forms of non-employment across early and mid-adulthood (Liao et al., 2022; Raab & Struffolino, 2022). The resulting clusters summarize broader patterns of labor-force attachment over time but do not directly measure job quality or the specific circumstances surrounding employment exits.

### 3.3 Epigenetic age acceleration and covariates

The 2016 HRS VBS includes 13 DNA methylation-based epigenetic clocks, from which we selected five commonly used measures: Horvath, Hannum, DunedinPACE, PhenoAge, and GrimAge (Crimmins & Kim, 2020; Simons et al., 2021). Horvath and Hannum are first-generation clocks (Crimmins et al., 2024; Hannum et al., 2013; Horvath, 2013) trained primarily to predict chronological age from DNA methylation patterns, whereas DunedinPACE, PhenoAge, and GrimAge are second-generation clocks designed to capture health-relevant biological aging, physiological dysregulation, pace of aging, or mortality risk (Levine, 2020; Lu et al., 2019). For each clock, we calculated epigenetic age acceleration (EAA) by regressing epigenetic age on chronological age and standardizing the residuals. This residualization isolates variation in epigenetic age or pace of aging independent of chronological age, while standardization allows effect sizes to be compared across clocks measured on different scales (Chen et al., 2016; Raffington & Belsky, 2022). Positive EAA values indicate an epigenetic age higher than expected given chronological age, whereas negative values indicate the reverse (Bernabeu et al., 2023).

#### 3.3.1 Covariates

Race/ethnicity was self-identified and categorized into three groups: non-Hispanic White (labeled as White [reference]), non-Hispanic Black (labeled as Black), and Hispanic/Latino (labeled as Hispanic). Sociodemographic and health-related covariates included gender, chronological age in 2016, marital status, years of education, self-reported early childhood health status, father's education, early childhood financial condition (with higher values indicating greater financial resources), number of chronic conditions in 2014, and nativity status. These covariates were selected to account for sociodemographic and early-life factors associated with employment trajectories and later-life biological aging.

### 3.4 Statistical analysis

#### 3.4.1 Sequences and cluster analyses

We first used optimal matching between sequences of spells (OMspell) to measure dissimilarities among employment histories. OMspell treats spells as the unit of comparison and accounts for both sequence order and spell duration, making it appropriate for employment histories in which the length of employment and non-employment spells is substantively meaningful (Abbott & Tsay, 2000; Billari & Piccarreta, 2005). Substitution and insertion-deletion costs were specified to distinguish transitions between employment and non-employment, between full- and part-time work, among non-employment states, and between the state before the first job record and other states. Full details on the cost matrix and OMspell parameters are provided in Supplementary Table S1. After calculating pairwise distances, we applied hierarchical clustering using Ward's method, a widely used approach in sequence analysis (Liao et al., 2022; Raab & Struffolino, 2022). A four-cluster solution was selected based on cluster quality indices and permutation tests (Supplementary Fig. S2). Sequence analyses were conducted in R using TraMineR and TraMineRextras.

We next examined racial and ethnic patterning in trajectory membership using survey-weighted multinomial logistic regression, with employment trajectory as the outcome and race/ethnicity as the primary independent variable, adjusting for all covariates. Then, we used survey-weighted OLS regression to estimate associations between employment trajectories and EAA across the five epigenetic clocks. To assess whether racial and ethnic differences in EAA varied across trajectories, we added race-by-trajectory interaction terms to the OLS models. From these models, we estimated adjusted marginal means and computed within-trajectory pairwise contrasts between racial and ethnic groups, applying Sidak corrections for multiple comparisons. As a sensitivity analysis, we additionally adjusted for broad U.S. Census occupational categories, with professional/managerial occupations as the reference group. Because occupational information was missing for some respondents, these models used a reduced analytic sample (N = 1520) and are presented in the supplement rather than as the primary specification. All analyses were conducted in R (version 4.5.1) using survey-weighted multinomial logistic and OLS regression models.

## 4 Results

### 4.1 Employment trajectories

Fig. 1 presents state distribution plots for the four employment trajectories identified through sequence and cluster analyses from ages 18 to 50. In the regression analytic sample, the Stable Full-Time trajectory (N = 932) includes respondents who entered full-time work early and remained continuously attached to full-time employment through midlife. The Late Workforce Entry trajectory (N = 150) is characterized by an extended pre-employment period into the late 20s and 30s, followed by gradual entry into paid work. The Multiple Job Disruption trajectory (N = 567) reflects more heterogeneous work histories, with full- and part-time employment interspersed with non-employment spells across adulthood. The Long-Term Non-Employment trajectory (N = 212) includes respondents with some early employment followed by increasing withdrawal from paid work beginning in the late 20s, with limited re-entry before age 50.

**Fig. 1:**
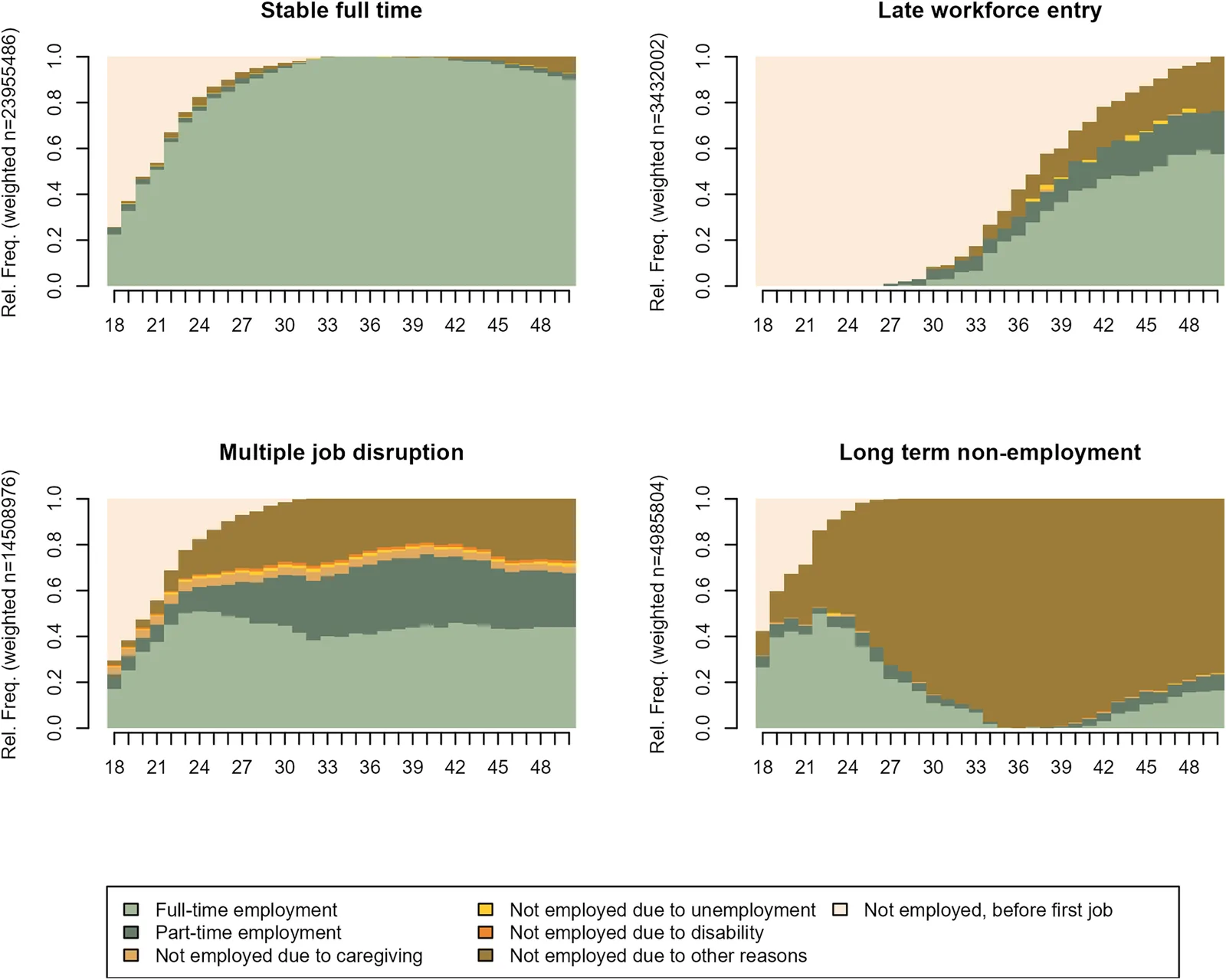
Employment trajectories between ages 18–50 (*N* = 2147). *Note.* Four stacked-area panels for four groups (Stable full time, Late workforce entry, Multiple job disruption, Long-term non-employment); colors denote status categories. y-axis = relative frequency; “weighted n” = weighted sample size. **Abbreviations:** Rel. Freq. = relative frequency; n = sample size.

### 4.2 Descriptive patterns of employment trajectories

Table 1 shows that employment trajectory membership differed significantly across racial and ethnic groups (p < .001). White respondents were more likely to follow the Stable Full-Time trajectory than Black or Hispanic respondents (52% vs. 45% and 39%, respectively; both p < .05). In contrast, Black and Hispanic respondents were more likely than White respondents to follow the Late Workforce Entry trajectory (13% and 15% vs. 6.7%). Differences were smaller for Multiple Job Disruption and Long-Term Non-Employment. These patterns indicate that racial and ethnic differences in employment histories were concentrated primarily in stable full-time attachment and delayed labor-force entry, rather than in chronic disruption alone.

**Table 1:** Descriptive statistics by race/ethnicity (N = 1861).

Characteristic	Overall N = 1,861^a^	White N = 1,496^a^	Black N = 210^a^	Hispanic N = 155^a^	p-value^b^
PhenoAge EAA	−0.01 (1.00)	−0.03 (0.99)	0.00 (1.04)	0.11 (1.01)	0.2
GrimAge EAA	−0.03 (0.98)	−0.07 (0.98)	0.23 (0.97)	0.02 (0.99)	<0.001
DunedinPACE EAA	−0.05 (0.98)	−0.09 (0.96)	0.23 (1.03)	−0.01 (0.97)	<0.001
Horvath EAA	0.00 (0.99)	0.02 (0.97)	0.06 (1.05)	−0.20 (1.01)	0.077
Hannum EAA	0.02 (0.99)	0.07 (0.99)	−0.35 (1.05)	0.04 (0.89)	<0.001
**Employment trajectory**					<0.001
Stable full time	932 (50%)	777 (52%)	94 (45%)	61 (39%)	
Late workforce entry	150 (8.1%)	100 (6.7%)	27 (13%)	23 (15%)	
Multiple job disruption	567 (30%)	454 (30%)	65 (31%)	48 (31%)	
Long term non-employment	212 (11%)	165 (11%)	24 (11%)	23 (15%)	
**Gender**					0.014
Male	813 (44%)	671 (45%)	72 (34%)	70 (45%)	
Female	1048 (56%)	825 (55%)	138 (66%)	85 (55%)	
**Marital status (2016)**					<0.001
Married	1199 (64%)	989 (66%)	104 (50%)	106 (68%)	
Divorced/separated	282 (15%)	211 (14%)	51 (24%)	20 (13%)	
Widowed	288 (15%)	233 (16%)	30 (14%)	25 (16%)	
Never married	92 (4.9%)	63 (4.2%)	25 (12%)	4 (2.6%)	
**Years of education**	13.70 (2.60)	13.98 (2.22)	13.52 (2.49)	11.26 (4.32)	<0.001
College or higher					<0.001
Less than college	1240 (67%)	964 (64%)	150 (71%)	126 (81%)	
College or higher	621 (33%)	532 (36%)	60 (29%)	29 (19%)	
**Age (2016)**	70 (9)	71 (9)	66 (7)	66 (8)	<0.001
**Nativity**					<0.001
US-born	1702 (91%)	1443 (96%)	195 (93%)	64 (41%)	
Foreign-born	159 (8.5%)	53 (3.5%)	15 (7.1%)	91 (59%)	
Labor force status (2016)					<0.001
Working	471 (25%)	342 (23%)	59 (28%)	70 (45%)	
Retired	1320 (71%)	1109 (74%)	141 (67%)	70 (45%)	
Not working	70 (3.8%)	45 (3.0%)	10 (4.8%)	15 (9.7%)	
**Number of chronic conditions** (2014)	2.10 (1.41)	2.10 (1.41)	2.32 (1.41)	1.83 (1.41)	0.002
**Childhood health status**					<0.001
Poor	10 (0.5%)	5 (0.3%)	2 (1.0%)	3 (1.9%)	
Fair	83 (4.5%)	59 (3.9%)	10 (4.8%)	14 (9.0%)	
Good	244 (13%)	176 (12%)	38 (18%)	30 (19%)	
Very Good	447 (24%)	370 (25%)	44 (21%)	33 (21%)	
Excellent	1077 (58%)	886 (59%)	116 (55%)	75 (48%)	
**Father's education**					<0.001
Low	848 (46%)	599 (40%)	134 (64%)	115 (74%)	
Middle	757 (41%)	661 (44%)	64 (30%)	32 (21%)	
High	256 (14%)	236 (16%)	12 (5.7%)	8 (5.2%)	
**Childhood financial condition**					<0.001
Poor	451 (24%)	309 (21%)	77 (37%)	65 (42%)	
Average	1258 (68%)	1060 (71%)	123 (59%)	75 (48%)	
Rich	152 (8.2%)	127 (8.5%)	10 (4.8%)	15 (9.7%)	

a Mean (SD); n (%).

b Kruskal-Wallis rank sum test; Pearson's Chi-squared test.

Epigenetic age acceleration also varied by race and ethnicity, especially on the second-generation clocks. Compared to White adults, Black adults showed faster DunedinPACE (0.23 vs. −0.09) and GrimAge acceleration (0.23 vs. −0.07; both p < .001), while PhenoAge did not differ across groups (p = .2). On the first-generation clocks, Black adults showed slower Hannum acceleration (−0.35 vs. 0.07, p < .001) and Hispanic adults showed slower Horvath acceleration (−0.20 vs. 0.02, p < .05).

Racial and ethnic groups also differed in socioeconomic, demographic, early-life, and health characteristics. Hispanic adults had the lowest average education and were much more likely to be foreign-born. Black adults were less likely to be married and more likely to be divorced, separated, or never married. Black and Hispanic adults also reported greater early-life disadvantage, including lower father's education and more childhood financial hardship, than White adults. Health and occupational differences followed similar patterns: Black and Hispanic adults reported worse self-rated health, and they were less likely than White adults to have held professional or managerial occupations and more likely to have worked in service occupations.

### **4.3** Racial and ethnic patterning of employment trajectories

Table 2 presents survey-weighted multinomial logistic models predicting employment trajectory membership, with Stable Full-Time as the reference trajectory (see Table 1). Racial and ethnic differences were concentrated in Late Workforce Entry. Compared with White adults, Black adults (OR = 2.26, p < .05) and Hispanic adults (OR = 2.45, p < .05) had higher odds of Late Workforce Entry, but they did not differ significantly in their odds of Multiple Job Disruption or Long-Term Non-Employment. Nativity was also strongly associated with delayed entry: U.S.-born adults had lower odds of Late Workforce Entry than foreign-born adults (OR = 0.26, p < .01). Together, these results suggest that racial and ethnic differences in employment trajectories were driven primarily by delayed labor-force entry rather than by mid-career disruption or sustained non-employment.

**Table 2:** Weighted multinomial logistic regression predicting employment trajectory membership (N = 1861).

Ref = Stable Full time (N = 932)	Late workforce entry (N = 150)	Multiple job disruption (N = 567)	Long term non-employment (N = 212)
	*Odds Ratios [95% CI]*	*Odds Ratios [95% CI]*	*Odds Ratios [95% CI]*
(Intercept)	0.024**	1.310	0.116
	[0.002, 0.350]	[0.204, 8.425]	[0.008, 1.628]
Female (ref = male)	6.593***	7.534***	9.345***
	[3.825, 11.365]	[5.543, 10.241]	[5.838, 14.960]
Race (ref = non-His White)			
Non-Hispanic Black	2.258*	1.135	1.151
	[1.109, 4.598]	[0.685, 1.881]	[0.561, 2.363]
Hispanic	2.453*	1.161	1.083
	[1.131, 5.322]	[0.615, 2.191]	[0.457, 2.565]
Marital status (ref = married)			
Divorced/separated	1.058	0.702+	0.521*
	[0.558, 2.007]	[0.470, 1.048]	[0.278, 0.975]
Widowed	1.263	1.049	1.313
	[0.689, 2.315]	[0.666, 1.651]	[0.818, 2.108]
Never married	2.230	1.090	0.872
	[0.782, 6.361]	[0.510, 2.329]	[0.293, 2.598]
Education (year)	0.975	0.964	0.883**
	[0.877, 1.082]	[0.903, 1.029]	[0.820, 0.951]
Age at 2016	1.038**	0.996	1.037**
	[1.011, 1.065]	[0.978, 1.015]	[1.010, 1.065]
Childhood health (higher better)	0.843	0.858+	0.784*
	[0.639, 1.111]	[0.737, 1.000]	[0.620, 0.992]
Father's education (ref = Low)			
Middle	0.889	0.886	0.807
	[0.539, 1.468]	[0.647, 1.214]	[0.514, 1.267]
High	0.540	1.006	0.557
	[0.235, 1.240]	[0.634, 1.596]	[0.269, 1.154]
Childhood financial condition (ref = Low)			
Average	1.353	1.065	1.448
	[0.760, 2.409]	[0.753, 1.506]	[0.837, 2.503]
Rich	1.097	1.042	1.312
	[0.386, 3.116]	[0.580, 1.872]	[0.566, 3.040]
US-born (ref: Foreign-born)	0.261**	0.666	0.773
	[0.115, 0.589]	[0.343, 1.290]	[0.304, 1.967]
N chronic conditions (2014)	1.035	0.987	0.853*
	[0.888, 1.208]	[0.881, 1.106]	[0.731, 0.997]


Num.Obs.	1861		
RMSE	2.12		

+ *p* < .1, *p < .05, **p < .01, ***p < .001.

Gender was strongly associated with trajectory membership. Compared with men, women had substantially higher odds of Late Workforce Entry (OR = 6.59), Multiple Job Disruption (OR = 7.53), and Long-Term Non-Employment (OR = 9.35; all p < .001), indicating a gendered structure of life-course employment histories. Other predictors were trajectory-specific. More years of education were associated with lower odds of Long-Term Non-Employment (OR = 0.88, p < .01), as was better childhood health (OR = 0.79, p < .05). Older age was associated with higher odds of Late Workforce Entry (OR = 1.04, p < .01) and Long-Term Non-Employment (OR = 1.04, p < .01). Father's education and childhood financial condition were not significantly associated with trajectory membership after adjustment.

### 4.4 Employment trajectories and epigenetic age acceleration

Racial and ethnic differences in epigenetic age acceleration were significant in the survey-weighted OLS models, even after adjustment for employment trajectory and sociodemographic covariates (Table 3, Model 1). Black adults showed faster DunedinPACE (β = 0.353, p < .01) and GrimAge EAA (β = 0.253, p < .01) than White adults, averaged across trajectories and adjusted for covariates. Black adults also showed lower Hannum EAA than White adults (β = −0.372, p < .001). Hispanic adults showed marginally lower Horvath EAA than White adults (β = −0.203, p < .10), but did not differ significantly on the other clocks. Employment trajectories, however, were largely unrelated to EAA after covariate adjustment. Relative to the Stable Full-Time trajectory, Late Workforce Entry, Multiple Job Disruption, and Long-Term Non-Employment did not differ significantly on any of the five clocks. Thus, racial and ethnic differences in EAA persisted after accounting for employment trajectory, while trajectory membership itself showed little independent association with EAA.

**Table 3:** Weighted OLS regression of epigenetic age acceleration on employment trajectories, by epigenetic clock (N = 1861).

	Model 1: Main Effects)					Model 2: Race × Trajectory Interaction				
	Horvath	Hannum	Dunedin	Pheno	GrimAge	Horvath	Hannum	Dunedin	Pheno	GrimAge
(Intercept)	0.413	0.688*	0.225	0.695+	1.347***	0.420	0.671+	0.210	0.642	1.332***
	[-0.247, 1.072]	[0.013, 1.363]	[-0.433, 0.882]	[-0.089, 1.479]	[0.707, 1.987]	[-0.243, 1.084]	[-0.005, 1.347]	[-0.447, 0.867]	[-0.140, 1.424]	[0.694, 1.970]
Late workforce entry	0.037	−0.156+	0.110	−0.082	0.131	0.066	−0.159+	0.121	−0.122	0.168
	[-0.168, 0.243]	[-0.319, 0.006]	[-0.114, 0.333]	[-0.302, 0.137]	[-0.073, 0.335]	[-0.179, 0.311]	[-0.344, 0.026]	[-0.148, 0.390]	[-0.368, 0.123]	[-0.065, 0.400]
Multiple job disruption	−0.054	−0.125+	−0.012	−0.123+	−0.055	−0.072	−0.170*	−0.000	−0.167*	−0.075
	[-0.183, 0.075]	[-0.255, 0.005]	[-0.136, 0.111]	[-0.260, 0.014]	[-0.176, 0.065]	[-0.210, 0.066]	[-0.308, −0.031]	[-0.132, 0.131]	[-0.314, −0.020]	[-0.203, 0.054]
Long term non-employment	−0.035	−0.132	−0.073	−0.069	−0.095	−0.067	−0.173+	−0.115	−0.109	−0.152+
	[-0.217, 0.147]	[-0.313, 0.049]	[-0.260, 0.113]	[-0.280, 0.142]	[-0.243, 0.053]	[-0.266, 0.132]	[-0.373, 0.027]	[-0.320, 0.090]	[-0.345, 0.127]	[-0.313, 0.008]
Black	0.036	−0.372***	0.353**	0.042	0.253**	0.008	−0.584***	0.431*	−0.150	0.191
	[-0.148, 0.219]	[-0.565, −0.179]	[0.135, 0.570]	[-0.143, 0.226]	[0.085, 0.422]	[-0.275, 0.291]	[-0.895, −0.272]	[0.095, 0.767]	[-0.418, 0.117]	[-0.069, 0.450]
Hispanic	−0.203+	−0.045	−0.029	0.005	−0.170	−0.342**	−0.159	−0.127	−0.180	−0.305
	[-0.419, 0.013]	[-0.279, 0.190]	[-0.264, 0.207]	[-0.241, 0.251]	[-0.418, 0.079]	[-0.588, −0.096]	[-0.375, 0.058]	[-0.472, 0.218]	[-0.492, 0.132]	[-0.672, 0.062]
Black × Late workforce entry						0.040	0.271	−0.119	0.238	−0.177
						[-0.568, 0.649]	[-0.333, 0.874]	[-0.700, 0.462]	[-0.353, 0.830]	[-0.734, 0.380]
Hispanic × Late workforce entry						−0.161	−0.054	0.038	0.321	−0.005
						[-0.628, 0.307]	[-0.442, 0.335]	[-0.583, 0.659]	[-0.421, 1.063]	[-0.723, 0.713]
Black × Multiple job disruption						0.000	0.429*	−0.247	0.442*	0.138
						[-0.382, 0.383]	[0.021, 0.837]	[-0.748, 0.253]	[0.037, 0.847]	[-0.234, 0.510]
Hispanic × Multiple job disruption						0.376+	0.333	0.077	0.324	0.205
						[-0.067, 0.819]	[-0.174, 0.841]	[-0.382, 0.536]	[-0.144, 0.792]	[-0.297, 0.707]
Black × Long term non-employment						0.228	0.480+	0.144	0.299	0.409*
						[-0.383, 0.839]	[-0.025, 0.985]	[-0.402, 0.690]	[-0.156, 0.755]	[0.009, 0.810]
Race × Hispanic × Long term non-employment						0.326	0.182	0.581+	0.413+	0.575*
						[-0.222, 0.874]	[-0.213, 0.578]	[-0.055, 1.216]	[-0.058, 0.884]	[0.024, 1.125]
Num.Obs.	1861	1861	1861	1861	1861	1861	1861	1861	1861	1861
R2	0.029	0.074	0.060	0.041	0.240	0.031	0.078	0.063	0.045	0.242
R2 Adj.	0.019	0.065	0.051	0.032	0.232	0.018	0.066	0.051	0.032	0.233
AIC	5156.6	5133.8	5141.1	5327.7	4799.7	5161.9	5135.1	5148.0	5331.0	4805.5
BIC	6543.7	6586.2	6651.2	6777.2	6568.9	6591.2	6625.9	6694.0	6820.2	6622.4

*Note.* Entries are weighted OLS coefficients with 95% confidence intervals in brackets. Outcome is epigenetic age acceleration (EAA) for each clock. Other covariate coefficients are omitted from the table for brevity; all models include the full set of covariates described in Section 3.3.1. Reference categories: Stable Full-Time (trajectory), White (race/ethnicity), male (sex), married (marital status), low (father's education), poor (childhood finances), foreign-born (nativity). All models apply HRS-VBS sampling weights. + p < .10, *p < .05, **p < .01, ***p < .001.

Race-by-trajectory interactions provided little evidence that employment trajectories moderated racial and ethnic differences in EAA (Table 3, Model 2). A small number of combined race-by-trajectory estimates reached nominal significance relative to White adults in Stable Full-Time. Hispanic adults in Late Workforce Entry had lower Horvath EAA (β = −0.436, p < .05) and lower Hannum EAA (β = −0.372, p < .05). Black adults in Multiple Job Disruption had lower Hannum EAA (β = −0.325, p < .05), while Black adults in Late Workforce Entry had higher DunedinPACE (β = 0.433, p < .05). Black adults in Long-Term Non-Employment also had higher DunedinPACE (β = 0.460, p < .05) and GrimAge EAA (β = 0.448, p < .01). However, these isolated estimates did not provide consistent evidence of moderation. Joint Wald tests showed that the full set of race-by-trajectory interaction terms was not significant for any clock: Horvath (F = 1.02, p = .409), Hannum (F = 1.27, p = .268), DunedinPACE (F = 0.86, p = .525), PhenoAge (F = 1.28, p = .263), or GrimAge (F = 1.58, p = .150). Given the number of contrasts tested and the non-significant joint tests, we do not interpret the nominally significant estimates as evidence that employment trajectories moderated racial and ethnic differences in EAA (RQ3). Occupation-adjusted sensitivity models yielded substantively similar conclusions: racial differences in DunedinPACE and GrimAge remained, and race-by-trajectory interactions remained jointly non-significant (Supplementary Table S2).

We then examined whether racial and ethnic differences in EAA persisted within trajectories (RQ4). Within-trajectory differences were most evident in the Stable Full-Time and Long-Term Non-Employment groups. In Stable Full-Time, racial and ethnic groups differed on Hannum (F = 7.21, p < .001), Horvath (F = 3.77, p < .05), and DunedinPACE (F = 3.56, p < .05). Pairwise contrasts showed that Black adults had lower Hannum EAA but higher DunedinPACE than White adults, while Hispanic adults had lower Horvath EAA than White adults (Fig. 2). In Long-Term Non-Employment, racial and ethnic groups differed on GrimAge (F = 7.72, p < .001) and DunedinPACE (F = 4.25, p < .05), with Black adults showing higher EAA than White adults on both stress-sensitive and health-relevant clocks. No within-trajectory differences were observed for PhenoAge.

**Fig. 2:**
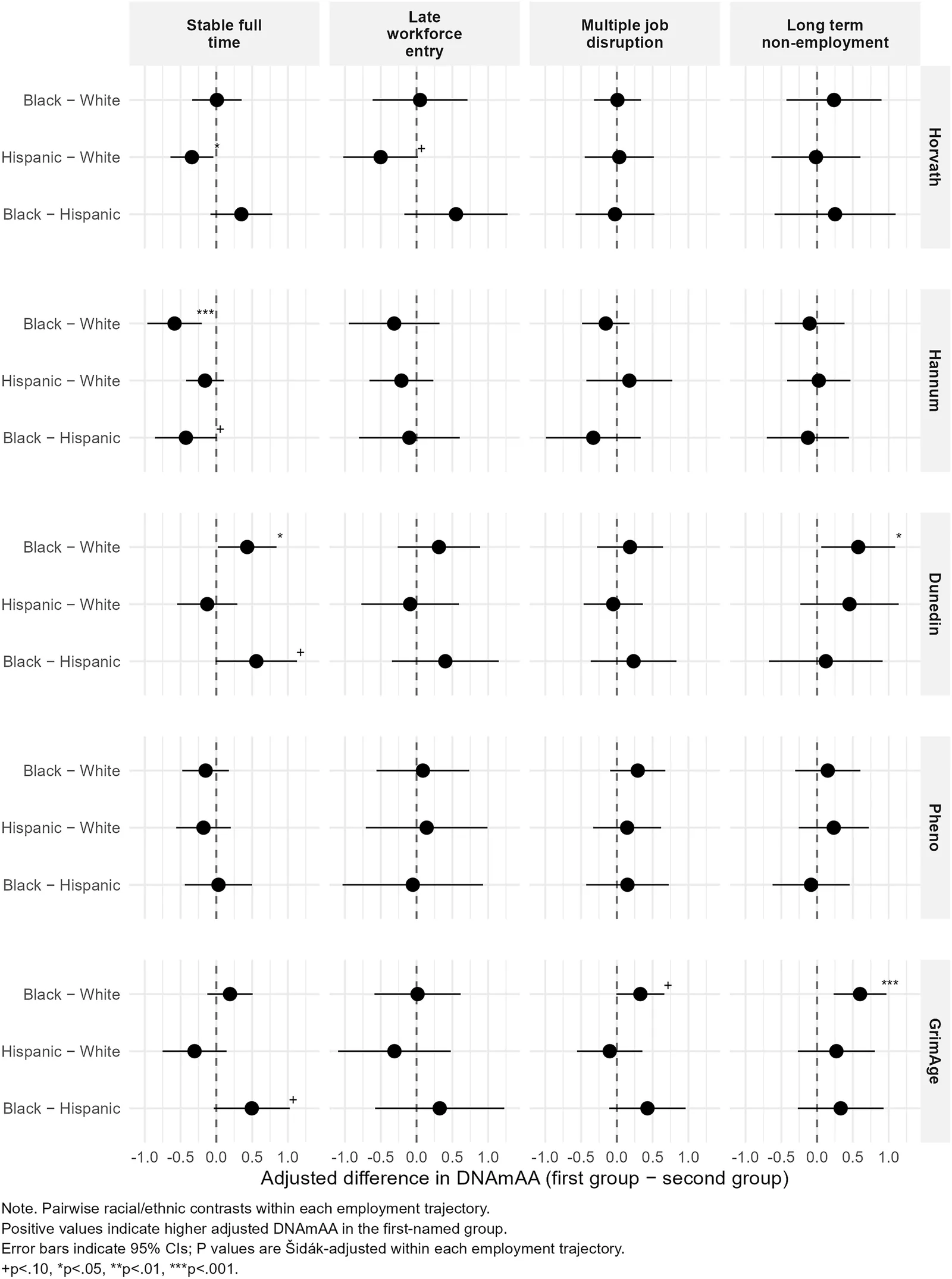
Pairwise race differences within employment-trajectory clusters across five epigenetic aging models. *Note.* Points indicate estimates; bars show 95% confidence intervals; dashed vertical line marks 0. Significance: ***, **, *, + (*p* < .001, 0.01, 0.05, 0.10). **Abbreviations:** nonHis = non-Hispanic.

## 5 Discussion

This study examined whether life-course employment trajectories were racially and ethnically patterned, whether they were associated with later-life EAA, and whether racial and ethnic differences in EAA persisted within similar work histories. Using sequence-based employment histories from the HRS, we identified four trajectories of labor-force attachment from ages 18 to 50 and linked them to five epigenetic clocks. Three findings stand out. First, employment trajectories were racially and ethnically patterned, with Black and Hispanic adults less likely than White adults to follow stable full-time work histories and more likely to enter the workforce late. Second, employment trajectories themselves were not strongly or consistently associated with EAA, nor did they significantly moderate racial and ethnic differences in EAA. Third, racial differences in EAA persisted within trajectories, including within Stable Full-Time work and Long-Term Non-Employment. Together, these findings suggest that similar employment trajectories did not necessarily correspond to similar biological aging across racial and ethnic groups.

By reconstructing complete employment trajectories rather than relying on point-in-time employment status, this study shows that racial and ethnic differences in work histories were most clearly expressed in stable full-time attachment and delayed labor-force entry. White adults were most likely to follow a Stable Full-Time trajectory, whereas Black and Hispanic adults were less likely to follow this pathway and more likely to enter the workforce late. Although prior research has documented greater employment instability, unemployment, and labor-market disadvantage among Black and Hispanic workers (Doede, 2016; Lipscomb et al., 2006; Weisshaar & Cabello-Hutt, 2020), adjusted models in this study did not show significant racial or ethnic differences in Multiple Job Disruption or Long-Term Non-Employment. Instead, the clearest pattern was delayed access to sustained employment. The meaning of Late Workforce Entry likely differed across groups. Among Black adults, who were predominantly U.S.-born and had higher average education than Hispanic adults in this sample, delayed entry is more consistent with structural barriers to stable employment, including discriminatory hiring and unequal access to protected full-time work (Burgard et al., 2009; Doede, 2016; Pager et al., 2009). Among Hispanic adults, delayed entry may partly reflect nativity, migration timing, educational pathways, and credentialing barriers.

Despite this racial and ethnic patterning, employment trajectories showed limited associations with EAA. This does not mean that employment histories are irrelevant for biological aging. Rather, sequence-based trajectories capture the timing, duration, and continuity of labor-force attachment, but not the job conditions embedded within those histories, including benefits, occupational hazards, job demands, job control, work strain, and working-time arrangements (Benach et al., 2014; Eisenberg-Guyot et al., 2020). Also, this distinction helps interpret the counterintuitive finding that Multiple Job Disruption was not associated with faster EAA among White adults and was marginally associated with slower aging on some clocks. Supplemental analyses showed that the composition of non-employment in this cluster differed by race and ethnicity: White adults' non-employment years were most often attributed to caregiving or family reasons (52.6%), Black adults' to unemployment (48.4%), and Hispanic adults’ to disability or medical leave (48.9%). These experiences may carry different implications for stress exposure, economic strain, work–family strain, financial stress and biological aging than unstable or precarious employment (Baranowska-Rataj & Matysiak, 2016; Brand, 2015; Meyer & Parker, 2011). The findings therefore shift the analytic focus from whether employment trajectories directly predict EAA to whether racial and ethnic differences in EAA persist within shared trajectories.

Within-trajectory comparisons provide the clearest evidence for this pattern. Black adults showed higher DunedinPACE than White adults within Stable Full-Time work and higher GrimAge and DunedinPACE within Long-Term Non-Employment. This distinction is relevant because continuous full-time employment is often viewed as a relatively advantaged employment pathway. In this study, however, stable full-time attachment did not equalize biological aging. This finding supports the argument that racialized labor-market inequality operates not only through sorting into different employment trajectories, but also through unequal conditions within similar trajectories (Doede, 2016; Lipscomb et al., 2006). Occupational composition offers one possible explanation. Supplemental analyses showed that Stable Full-Time work was itself racially patterned. Specifically, White adults in this trajectory were more often in professional and managerial occupations (38%), whereas Black adults were more often in service and sales positions (53%), so the same trajectory label did not represent the same kind of work (Supplementary Table S2). Sensitivity models also showed that service and manual occupations were associated with faster GrimAge aging, consistent with prior studies linking service, clerical, and manual work to accelerated biological aging (Andrasfay, Crimmins, et al., 2023; Assari, 2025; George et al., 2021). Yet adjustment for broad occupational categories did not eliminate Black–White differences in DunedinPACE and GrimAge, suggesting that occupational sorting captures only part of the racialized conditions embedded within stable work.

A similar pattern appeared within Long-Term Non-Employment, where Black adults experienced faster EAA on GrimAge and DunedinPACE clocks than White adults. White adults in this trajectory had more often previously held professional, managerial, sales, or office positions (roughly three-quarters) and were overwhelmingly female (85%). This composition may be consistent with more resourced exits related to homemaking or caregiving, which have been linked to reduced work–family strain and greater social support (Heshmati et al., 2023). Black adults, by contrast, were more concentrated in service and production work and included a larger share of men, suggesting that exits may have been more often tied to job loss, displacement, disability, or constrained labor-market opportunities. Such forms of non-employment can bring economic hardship, loss of health and pension benefits, and chronic stress, all of which may contribute to physiological wear and accelerated biological aging (Andrasfay, Crimmins, et al., 2023; Brand, 2015; Ervasti et al., 2017). Although we could not directly distinguish voluntary from involuntary non-employment, the divergent profiles suggest that similar employment trajectories may have different implications for biological aging depending partly on the reasons for labor-force exit, which may vary by race and ethnicity.

The clock-specific pattern of findings also warrants attention. Black adults showed lower Hannum EAA than White adults, including within Stable Full-Time work, even as they showed higher DunedinPACE and GrimAge in key comparisons. This pattern is not necessarily contradictory because epigenetic clocks capture different dimensions of aging. First-generation clocks such as Hannum and Horvath were trained primarily to predict chronological age, whereas GrimAge and DunedinPACE were designed to capture health-relevant biological aging, mortality risk, and pace of aging more directly (Levine et al., 2018; Lu et al., 2019; Raffington & Belsky, 2022). This interpretation is consistent with prior work showing limited socioeconomic patterning on first-generation clocks and mixed evidence linking employment instability to EAA (Freni-sterrantino et al., 2022; Hughes et al., 2018). Whether social inequalities in biological aging are detected may therefore depend in part on clock choice.

Taken together, these findings partially align with the weathering hypothesis by showing that racial differences in biological aging were not fully accounted for by broad employment trajectories. Black adults showed higher EAA on health-relevant clocks even within work histories that appeared similar in timing and continuity, suggesting that labor-force attachment alone does not capture the biological costs of racialized disadvantage. This pattern is consistent with evidence that structural racism and repeated exposure to disadvantage shape health across the life course, and that Black adults may receive weaker health returns from comparable socioeconomic and occupational standing (Assari, 2025; Geronimus et al., 2006; Williams et al., 2019). Employment trajectories therefore matter not because they fully explain racial differences in EAA, but because they reveal where those differences persist despite apparently similar life-course employment trajectories.

Several limitations should be noted. First, employment histories from ages 18 to 50 were reconstructed from retrospective reports and may be affected by recall error, especially for jobs held decades earlier or for short-term, informal, or intermittent employment. Second, the trajectories captured broad patterns of labor-force timing and stability, but not the work conditions embedded within them, such as wages, job security, benefits, occupational demands, workplace discrimination, or exposure to hazards. Although exit reasons were available, they were incorporated into broader trajectory groups, and “other” reasons for non-employment could not be further distinguished. As a result, a single trajectory may include exits with different health implications, including caregiving, homemaking, job loss, displacement, or health-related withdrawal. Third, some within-trajectory racial and ethnic comparisons were based on small cells, especially in Long-Term Non-Employment and in supplemental occupational and gender breakdowns; these findings should therefore be interpreted as descriptive. Fourth, the epigenetic measures were cross-sectional, so we could not estimate change in biological aging over time or fully rule out reverse causation from earlier health to both employment histories and later EAA. In addition, the non-random VBS DNA methylation sample may be subject to selective survival and participation. Respondents with the most adverse employment histories or cumulative stress exposures may have been less likely to survive, remain able to participate, or provide a blood sample by 2016. Because such selection can occur within demographic groups, sample diversity alone does not eliminate this concern and may have attenuated observed associations and disparities. Despite these limitations, this study integrates life-course employment histories, multiple epigenetic clocks, and an explicit focus on racial and ethnic disparities. The findings suggest that employment trajectories are useful markers of labor-force attachment, but only partly capture the racialized conditions through which work histories become biologically embedded. Future research with longitudinal biomarker data and more detailed measures of job quality, work exits, and workplace exposures would help clarify these mechanisms.

## 6 Conclusion

This study shows that life-course employment trajectories were patterned by race and ethnicity: Black and Hispanic adults were less likely than White adults to follow stable full-time work histories and more likely to enter the labor force late. Employment trajectories themselves, however, did not consistently predict EAA or account for racial and ethnic differences in it. Instead, Black–White differences persisted within trajectories, including within stable full-time work, and were most evident on GrimAge and DunedinPACE, clocks tied to mortality risk and pace of aging. These findings partially align with the weathering hypothesis by suggesting that racial differences in biological aging do not arise mainly through sorting into unstable employment pathways. Rather, they point to unequal conditions within apparently similar work histories, where the benefits of employment stability may not be equally realized across racial and ethnic groups.

## CRediT authorship contribution statement

**Wen-Hua Lai:** Writing – original draft, Investigation, Funding acquisition, Conceptualization. **Ting-Syuan Lin:** Writing – original draft, Visualization, Methodology, Formal analysis. **Tsai-Chin Cho:** Writing – review & editing, Data curation. **Helen C.S. Meier:** Writing – review & editing, Supervision. **Farah Ammous:** Writing – review & editing. **Amanda N. Leggett:** Writing – review & editing, Supervision, Conceptualization.

## Ethical statement

This study utilized publicly available, secondary data from the Health and Retirement Study (HRS). As no primary data collection was conducted and the data contain no identifiable information, additional ethical approval was not required for this analysis.

## Funding statement

This research was supported in part by the National Institute on Aging (P30 AG012846) through pilot funding from the Michigan Center on the Demography of Aging awarded to Dr. Lai. Dr. Meier is also supported by the National Institute on Aging (R01 AG080401).

## Conflict of interest

The authors declare that they have no known competing financial interests or personal relationships that could have appeared to influence the work reported in this paper.

## Data Availability

The data are publicly available through the Health and Retirement Study website: https://hrs.isr.umich.edu/data-products. Analysis code is available upon request from the corresponding author.
