# Supplementary material for: Employment trajectories and biological aging: Racial and ethnic disparities in work instability and epigenetic age acceleration
Source: SSM Popul Health. 2026 Sep 23;36:101973. doi: 10.1016/j.ssmph.2026.101973 (PMC13657233; doi:10.1016/j.ssmph.2026.101973)
Supplement: Supplementary file 1 [file mmc1.docx]

4 **Appendi**x

**Supplementary Tab1e S1**. Substitution-Cost Matrix for Employment-State Transitions

|  | FT | PT | CG | UE | DIS | OTH | PRE |
| --- | --- | --- | --- | --- | --- | --- | --- |
| FT | 0 | 1 | 4 | 4 | 4 | 4 | 3 |
| PT | 1 | 0 | 4 | 4 | 4 | 4 | 3 |
| CG | 4 | 4 | 0 | 1 | 1 | 1 | 2 |
| UE | 4 | 4 | 1 | 0 | 1 | 1 | 2 |
| DIS | 4 | 4 | 1 | 1 | 0 | 1 | 2 |
| OTH | 4 | 4 | 1 | 1 | 1 | 0 | 2 |
| PRE | 3 | 3 | 2 | 2 | 2 | 2 | 0 |

*Note.* FT = Full-time employment; PT = Part-time employment; CG = Not employed due to caregiving; UE = Not employed due to unemployment; DIS = Not employed due to disability or medical leave; OTH = Not employed due to other reasons; PRE = Not employed, years before the first job record.

**Supplementary Table S2. Race, Employment-Trajectory, and Occupation Coefficients With and Without Adjustment for Occupation**

|  | **Without occupation (N = 1,861)** | | | | | **With occupation (N = 1,520)** | | | | |
| --- | --- | --- | --- | --- | --- | --- | --- | --- | --- | --- |
| **Predictor** | **Horvath** | **Hannum** | **Dunedin**  **PACE** | **Pheno**  **Age** | **Grim**  **Age** | **Horvath** | **Hannum** | **DunedinPACE** | **Pheno**  **Age** | **GrimAge** |
| ***Model 1: Main effects*** | | | | | | | | | | |
| Non-Hispanic Black (ref: White) | 0.036 | -0.372*** | 0.353** | 0.042 | 0.253** | 0.029 | -0.425*** | 0.394** | -0.004 | 0.283** |
| Hispanic (ref: White) | -0.203+ | -0.045 | -0.029 | 0.005 | -0.170 | -0.280* | -0.096 | -0.015 | -0.052 | -0.178 |
| Late Workforce Entry (ref: Stable FT) | 0.037 | -0.156+ | 0.110 | -0.082 | 0.131 | 0.107 | -0.169+ | 0.179 | 0.008 | 0.186+ |
| Multiple Job Disruption (ref: Stable FT) | -0.054 | -0.125+ | -0.012 | -0.123+ | -0.055 | -0.062 | -0.144* | -0.061 | -0.137+ | -0.107+ |
| Long-Term Non-Employment (ref: Stable FT) | -0.035 | -0.132 | -0.073 | -0.069 | -0.095 | -0.053 | -0.258+ | -0.149 | -0.036 | -0.139 |
| ***Occupation (with-occupation model only; ref: professional/managerial)*** | | | | | | | | | | |
| Service | — | — | — | — | — | 0.026 | 0.108 | 0.113 | -0.027 | 0.143 |
| Sales and office | — | — | — | — | — | 0.041 | 0.065 | 0.015 | 0.002 | -0.019 |
| Natural resources/construction/maintenance | — | — | — | — | — | -0.149 | -0.193+ | 0.139 | -0.095 | 0.249* |
| Production/transportation | — | — | — | — | — | -0.113 | -0.206+ | 0.144 | -0.142 | 0.106 |
| Military | — | — | — | — | — | -0.001 | -0.100 | 0.048 | 0.155 | -0.052 |
| ***Model 2: Race × trajectory interaction*** | | | | | | | | | | |
| Black × Late Workforce Entry | 0.040 | 0.271 | -0.119 | 0.238 | -0.177 | 0.238 | 0.307 | -0.053 | 0.282 | -0.035 |
| Hispanic × Late Workforce Entry | -0.161 | -0.054 | 0.038 | 0.321 | -0.005 | -0.279 | -0.157 | 0.071 | 0.503 | 0.002 |
| Black × Multiple Job Disruption | 0.000 | 0.429* | -0.247 | 0.442* | 0.138 | -0.083 | 0.442* | -0.130 | 0.483* | 0.286 |
| Hispanic × Multiple Job Disruption | 0.376+ | 0.333 | 0.077 | 0.324 | 0.205 | 0.337 | 0.408 | 0.184 | 0.338 | 0.236 |
| Black × Long-Term Non-Employment | 0.228 | 0.480+ | 0.144 | 0.299 | 0.409* | 0.093 | 0.126 | 0.045 | -0.079 | 0.451 |
| Hispanic × Long-Term Non-Employment | 0.326 | 0.182 | 0.581+ | 0.413+ | 0.575* | 0.182 | 0.281 | 0.440 | 0.384 | 0.522+ |

*Note.* Weighted OLS coefficients for epigenetic age acceleration (EAA) on each clock. The left panel reproduces the main models (N = 1,861). The right panel adds U.S. Census occupational categories (reference = professional and managerial), together with 2016 labor-force status and 2014 self-rated health and chronic conditions; listwise deletion of missing occupational codes reduces N to 1,520. Occupational coefficients are shown only for the with-occupation model (dashes indicate not estimated in the left panel). Full covariate sets were estimated but other covariates are not displayed. Reference categories: Stable Full-Time, White, foreign-born. All models apply HRS-VBS sampling weights. + p < .10, * p < .05, ** p < .01, *** p < .001.

**Supplementary Figure S1.** Analytic sample derivation from the linked HRS–LHMS–VBS sample

**Supplementary Figure S2.** Cluster quality indices by number of clusters

*Note.* Lines show standardized values across cluster solutions for the point–biserial coefficient (PBC), Hubert’s Gamma (HG), and average silhouette width (ASW). Values in parentheses in the legend indicate the mean/SD z-score across *k*.

.
